# Mechanisms of the Incretin Effect in Subjects with Normal Glucose Tolerance and Patients with Type 2 Diabetes

**DOI:** 10.1371/journal.pone.0073154

**Published:** 2013-09-03

**Authors:** Andrea Mari, Jonatan I. Bagger, Ele Ferrannini, Jens J. Holst, Filip K. Knop, Tina Vilsbøll

**Affiliations:** 1 Institute of Biomedical Engineering, National Research Council, Padova, Italy; 2 Diabetes Research Division, Gentofte Hospital, University of Copenhagen, Copenhagen, Denmark; 3 Department of Internal Medicine, University of Pisa School of Medicine, Pisa, Italy; 4 The NNF Center for Basic Metabolic Research, Department of Biomedical Sciences, Panum Institute, University of Copenhagen, Copenhagen, Denmark; University of Las Palmas de Gran Canaria, Spain

## Abstract

The incretin effect on insulin secretion was investigated in 8 subjects with type 2 diabetes (T2D) and 8 with normal glucose tolerance (NGT), using 25, 75, and 125 g oral glucose tolerance tests (OGTT) and isoglycemic intravenous glucose infusions (IIGI). The ß-cell response was evaluated using a model embedding a dose-response (slope = glucose sensitivity), an early response (rate sensitivity), and potentiation (time-related secretion increase). The incretin effect, as OGTT/IIGI ratio, was calculated for each parameter. In NGT, the incretin effect on total secretion increased with dose (1.3±0.1, 1.7±0.2, 2.2±0.2 fold of IIGI, *P*<0.0001), mediated by a dose-dependent increase of the incretin effect on glucose sensitivity (1.9±0.4, 2.4±0.4, 3.1±0.4, *P* = 0.005), and a dose-independent enhancement of the incretin effect on rate sensitivity (894 [1145], 454 [516], 783 [1259] pmol m^−2^ L mmol^−1^ above IIGI; median [interquartile range], *P*<0.0001). The incretin effect on potentiation also increased (0.97±0.06, 1.45±0.20, 1.24±0.16, *P*<0.0001). In T2D, the incretin effect on total secretion (1.0±0.1, 1.1±0.1, 1.3±0.1, *P* = 0.004) and glucose sensitivity (1.2±0.2, 1.3±0.2, 2.0±0.2, *P* = 0.005) were impaired *vs* NGT; however, the incretin effect on rate sensitivity increased already at 25 g (269 [169], 284 [301], 193 [465] pmol m^−2^ L mmol^−1^ above IIGI; negligible IIGI rate sensitivity in T2D prevented the calculation of the fold increment). OGTT did not stimulate potentiation above IIGI (0.94±0.04, 0.89±0.06, 1.06±0.09; *P*<0.01 *vs* NGT). In the whole group, the incretin effect was inversely associated with total secretion during IIGI, although systematically lower in T2D. In conclusion, 1) In NGT, glucose sensitivity and potentiation mediate the dose-dependent incretin effect increase; 2) In T2D, the incretin effect is blunted *vs* NGT, but rate sensitivity is enhanced at all loads; 3) Relatively lower incretin effect in NGT is associated with higher secretion during IIGI, suggesting that the reduced incretin effect does not result from ß-cell dysfunction.

## Introduction

In subjects with normal glucose tolerance (NGT), the insulin response to the ingestion of a glucose load or a meal is influenced by the concomitant release of incretin hormones, glucagon-like peptide-1 (GLP-1) and glucose-dependent insulinotropic peptide (GIP), which potentiate glucose-induced insulin secretion. The incretin effect is defined as the potentiation of glucose-induced insulin secretion exerted by these hormones. The incretin effect has been shown to be reduced in patients with type 2 diabetes (T2D) [1], in prediabetic states such as impaired glucose tolerance and in obese, insulin resistant subjects with NGT [2], [3]. Historically, the incretin effect has been evaluated by comparing the insulin secretory response to an oral glucose tolerance test (OGTT) with that elicited by an isoglycemic intravenous (i.v.) glucose infusion (IIGI) (*i.e.*, at matched plasma glucose levels) [4]. Using this approach, it has been shown that in NGT subjects the incretin effect increases with the glucose load [4], and that this increase is diminished in T2D [5].

To quantify the incretin effect, these studies typically use the ratio of total insulin secretion during the OGTT to that obtained during the IIGI. This simple and robust index, however, does not provide detailed insight into the mechanisms by which the incretin response affects the dynamic relationship between insulin secretion and glucose concentrations. In this respect, studies using a mathematical model of insulin secretion [2], [6] have shown that, compared to i.v. glucose, an oral glucose load produces an upward shift of the ß-cell dose-response, as is also the case with an exogenous infusion of GLP-1 [7], [8]. In addition, a marked increase of the early insulin secretion response has also been observed with oral compared to i.v. glucose [2], [6].

In the present study, we assessed the impact of the incretin effect at ascending glucose doses on the ß-cell response characteristics of subjects with NGT or T2D, using the insulin secretion model employed previously [9], [10]. In addition, taking advantage of the multiple doses [5], we aimed at investigating the mechanisms underlying incretin effect regulation in NGT and T2D.

## Subjects and Methods

This study is based on data previously published by Bagger *et al.* [5]. A detailed description of the methods can be found in the original paper; this section gives a summary of the procedures.

### Subjects

Eight patients with T2D and 8 subjects with NGT and negative family history of diabetes were studied. Subjects and patients were matched with respect to sex, age, and body mass index (BMI) (sex: 3 males; age 57±11 years; BMI 29±3 kg/m^2^ in both groups; mean±SD). Patients with T2D were diagnosed according to the WHO criteria [11], the average duration of diabetes was 8 (6 to 36) months, all were treated with diet and exercise only, and had no evidence of vascular complications.

### Ethics Statement

All subjects agreed to participate after receiving oral and written information. The original protocol was approved by the Scientific-Ethical Committee of the Capital Region of Denmark and the Danish Data Protection Agency, and registered at www.ClinicalTrials.gov (NCT00529048).

### Experimental Methods

On six different days, each participant received, 25, 75, or 125 g of glucose orally, and three corresponding IIGIs over a 4-week period. On all occasions, subjects were studied in the morning after an overnight (10 hours) fast including medication and use of tobacco. On OGTT days (with glucose doses administered in random order), a cannula was inserted into a cubital vein for collection of arterialized blood samples. The cannulated forearm was placed in a heating box (50°C) throughout the experiment. Participants ingested 25 g, 75 g, or 125 g of water-free glucose, dissolved in 300 ml of water. Blood samples were drawn at baseline and then frequently for 240 min after glucose ingestion, for the measurement of plasma glucose, insulin, C-peptide, GLP-1, and GIP concentrations.

After the three OGTTs, three corresponding IIGIs were performed in randomized order. On the IIGI days, cannulas were inserted into cubital veins in both arms: one for collection of arterialized blood samples and one for glucose infusion. During the IIGI, the glucose infusion rate was adjusted to match the plasma glucose profile observed during the corresponding OGTT.

### Assays

Plasma glucose concentrations were measured by the glucose oxidase method using a glucose analyzer (Yellow Springs Instrument model 2300 STAT plus analyzer; YSI Inc., Yellow Springs, OH). Plasma insulin and C-peptide concentrations were measured using a two-sided electrochemiluminescence immunoassay (Roche/Hitachi Modular analytics; Roche Diagnostic GmbH, Mannheim, Germany) [12]. Plasma concentrations of total GLP-1 and GIP were measured by RIA, as previously described [13], [14].

### ß-cell Function Modeling

ß-cell function was assessed from the OGTT and IIGI using a model describing the relationship between insulin secretion and glucose concentration, which has been illustrated in detail previously [9], [10]. The model expresses insulin secretion (pmol^.^min^−1.^m^−2^) as the sum of two components. The first component represents the dependence of insulin secretion on absolute glucose concentration at any time point during the test through a dose-response function relating the two variables. Two parameters of the dose-response were calculated, the mean slope over the observed glucose range, denoted as ß-cell glucose sensitivity, and insulin secretion at a fixed reference glucose concentration, close to the basal value (5.3 mmol/L in NGT, 7.7 mmol/L in T2D). The dose-response is modulated by a potentiation factor, which accounts for the fact that during an acute stimulation insulin secretion is higher during the late phase of hyperglycemia than at the same glucose concentration on the early phase. As such, the potentiation factor encompasses several potentiating mechanisms (prolonged exposure to hyperglycemia, non-glucose substrates, gastro-intestinal hormones, neural modulation). It is set to be a positive function of time, and is constrained to average unity during the experiment. In NGT subjects, the potentiation factor typically increases from baseline to the end of a 2-hour OGTT [15]. To quantify the potentiation factor excursion, the ratio between the mean value in the last 3 hours and the baseline value (mean potentiation ratio) was calculated. The second insulin secretion component represents the dependence of insulin secretion on the rate of change of glucose concentration. This component is termed derivative component, and is determined by a single parameter, denoted as rate sensitivity. Rate sensitivity is a marker of early insulin release [15].

The model parameters were estimated both in the OGTTs and IIGIs from glucose and C-peptide concentrations by regularized least-squares, as previously described [9], [10]. Regularization involves the choice of smoothing factors, which were selected to obtain glucose and C-peptide model residuals with standard deviations close to the expected measurement error (∼1% for glucose and ∼4% for C-peptide). Insulin secretion rates were calculated from the model every 5 min. The integral of insulin secretion during the 4-hour tests was denoted as *total insulin secretion*.

### Calculations

Mean concentrations during the tests were computed by dividing the area under the curve by the test duration (4 hours). The total incretin effect was expressed as the ratio of total insulin secretion during the OGTT to that measured during the corresponding IIGI. An analogous incretin effect index was calculated using the ratio of ß-cell glucose sensitivity during OGTT and IIGI.

Insulin sensitivity was estimated from the OGTT using the Oral Glucose Insulin Sensitivity index (OGIS) [16].

### Statistical Methods

Normality of parameter distribution was tested by the Kolmogorov-Smirnov test. Parameters are presented as mean±SE (or median [interquartile range] when non-normally distributed). Group differences were tested by 3-way ANOVA for doubly repeated measures, with test (OGTT vs IIGI, repeated), dose (25, 75, or 125 g of glucose, repeated), and group (NGT vs T2D) as factors; all interaction terms were calculated. The dose dependence for insulin secretion at a fixed reference glucose concentration was tested separately for NGT and T2D using repeated measures ANOVA, as reference glucose levels were different in the two groups. For the incretin effects (expressed as the ratio of OGTT to IIGI parameters) and for OGIS, two-way ANOVA for repeated measures was used, with dose and group as factors. In these ANOVA models, variables with skewed distribution were log-transformed. The dose dependence for rate sensitivity and the change from IIGI to OGTT were tested separately for NGT and T2D using Friedman’s test, as the distribution of this parameter could not be normalized by log-transformation. For dose-unrelated variables, differences between groups were tested using the Wilcoxon rank sum test. Univariate associations were tested with the Spearman’s correlation coefficient (ρ); standard linear regression was used for multivariate associations. A *P*-value of 0.05 was considered statistically significant.

## Results

### Glucose Levels and Insulin Secretion

Mean glucose concentrations during the OGTT increased with the glucose dose in NGT (by ∼10% for the 75-g and ∼25% for the 125-g dose, compared to the 25-g dose) as well as T2D (∼45% for the 75-g and ∼55% for the 125-g dose) (*P*<0.0001 for the dose effect), and were higher in T2D than NGT (*P*<0.0001 for the group effect). In both groups, glucose levels were well matched during IIGI (Tables 1 & 2). Total insulin secretion also increased with the glucose dose in both NGT and T2D (*P*<0.0001); the increase was larger on the OGTT than the IIGI only in NGT (*P* = 0.004 for the interaction test × group).

**Table 1:** Metabolic and β-cell function parameters (mean±SE or median [interquartile range])*.

	IIGI			OGTT		
	25 g	75 g	125 g	25 g	75 g	125 g
**NGT**						
Fasting glucose (mmol/L)	5.3±0.1	5.3±0.2	5.5±0.1	5.3±0.2	5.4±0.1	5.5±0.1
Mean glucose (mmol/L)	6.0±0.2	6.5±0.3	7.2±0.3	5.7±0.2	6.4±0.3	7.4±0.3
Total insulin secretion (nmol/m^2^)	25 [14]	35 [25]	46 [26]	35 [12]	62 [25]	115 [47]
Glucose sensitivity (pmol^.^min^−1.^m^−2.^L^.^mmol^−1^)	39 [22]	41 [30]	34 [24]	68 [40]	93 [23]	116 [31]
Rate sensitivity (pmol^.^m^−2.^L^.^mmol^−1^)	388 [257]	348 [383]	426 [310]	1298 [1030]	826 [706]	1168 [1739]
Mean potentiation	0.9 [0.1]	1.0 [0.3]	1.2 [0.5]	0.9 [0.2]	1.5 [0.5]	1.5 [0.4]
Mean GLP-1 (pmol/L)	13 [10]	18 [3]	20 [4]	15 [7]	29 [14]	36 [25]
Mean GIP (pmol/L)	5 [8]	8 [8]	8 [8]	22 [17]	51 [35]	83 [51]
**T2D**						
Fasting glucose (mmol/L)	7.8±0.4	8.0±0.4	7.6±0.2	7.7±0.3	7.9±0.2	7.5±0.2
Mean glucose (mmol/L)	8.8±0.4	12.8±0.6	13.6±0.8	8.7±0.4	12.6±0.5	13.6±0.8
Total insulin secretion (nmol/m^2^)	39 [13]	60 [26]	72 [31]	42 [22]	59 [33]	83 [26]
Glucose sensitivity (pmol^.^min^−1.^m^−2.^L^.^mmol^−1^)	15 [7]	15 [7]	15 [6]	16 [6]	19 [10]	30 [15]
Rate sensitivity (pmol^.^m^−2.^L^.^mmol^−1^)	0 [52]	0 [7]	0 [0]	289 [137]	284 [335]	219 [465]
Mean potentiation	1.2 [0.1]	1.4 [0.1]	1.4 [0.2]	1.2 [0.2]	1.3 [0.2]	1.3 [0.6]
Mean GLP-1 (pmol/L)	16 [8]	18 [4]	17 [10]	19 [10]	26 [7]	36 [20]
Mean GIP (pmol/L)	8 [9]	6 [8]	7 [11]	25 [12]	44 [15]	53 [23]

* Statistical significance is shown in Table 2. Fasting glucose and incretin hormones have been reported previously in Ref. [5].

**Table 2:** Statistical significance of differences by test, dose, and group (ANOVA for doubly repeated measures)*.

Variable	Test	Dose	Group	Test *x* Dose	Test *x* Group	Group *x* Dose
Fasting glucose	ns	ns	0.0001	ns	ns	ns
Mean glucose	ns	0.0001	0.0001	ns	ns	0.0001
Total insulin secretion	0.0001	0.0001	ns	ns	0.004	ns
Glucose sensitivity	0.0001	0.005	0.0001	0.013	0.005	ns
Mean potentiation	ns	0.0001	ns	ns	ns	0.015
Mean GLP-1	0.0001	0.0001	ns	0.003	ns	ns
Mean GIP	0.047	ns	ns	ns	ns	ns

* Test = OGTT *vs* IIGT; Dose = 25 *vs* 75 *vs* 125 g of glucose orally; Group = NGT *vs* T2D.

### ß-cell Function

**Figure 1:**
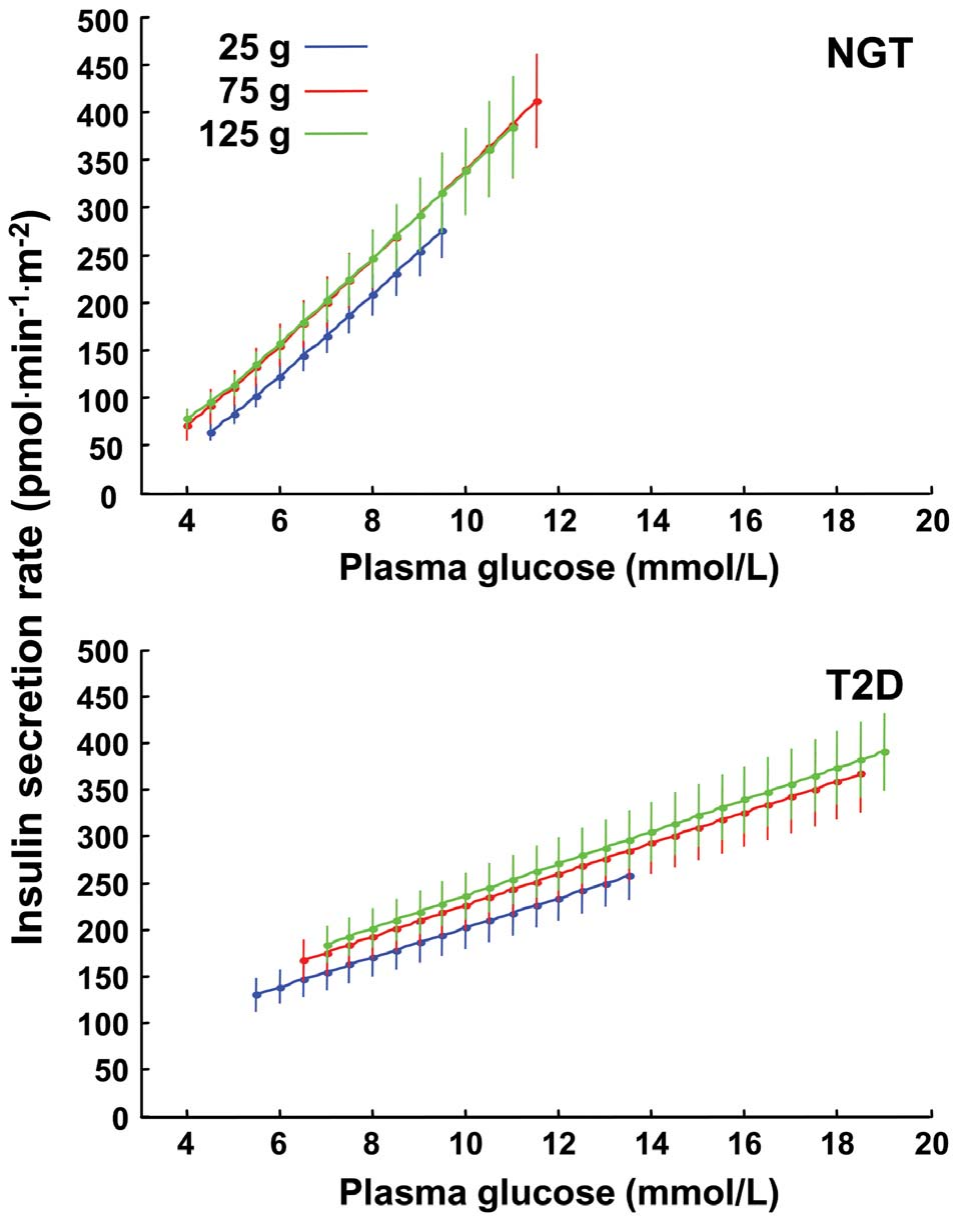
Beta-cell dose-response. Insulin secretion dose-response obtained from the IIGI at the three glucose doses in subjects with NGT subjects (*top*) and patients with T2D (*bottom*). Plots are mean±SE; glucose spans reflect the average observed levels.

**Figure 2:**
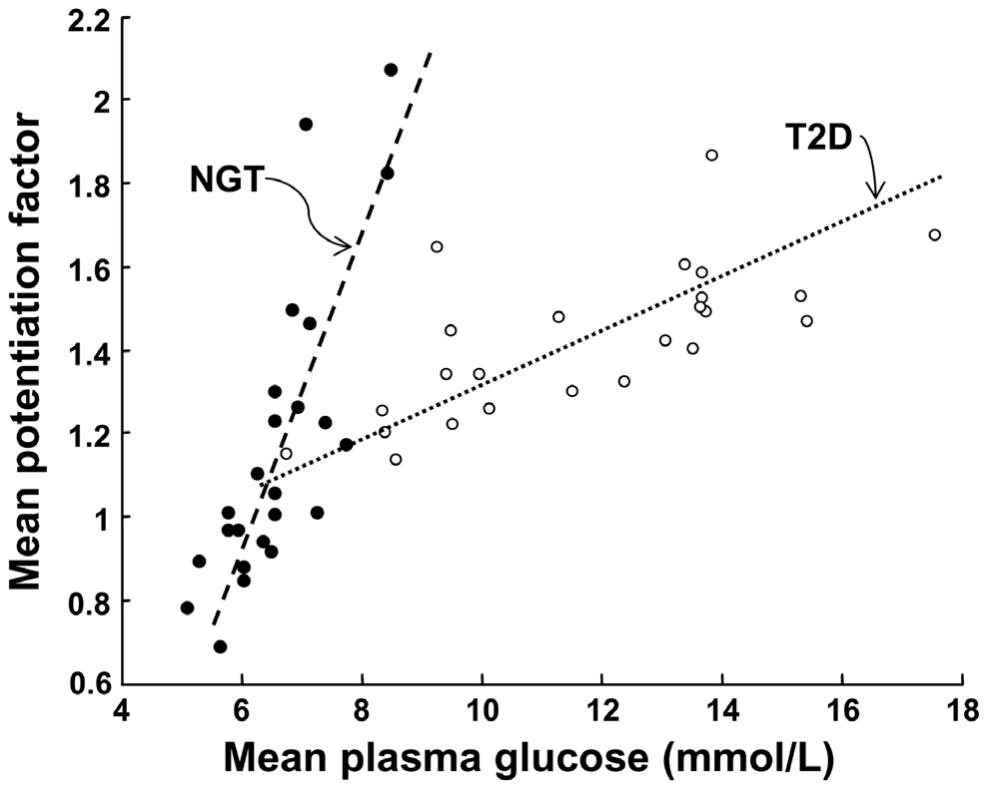
Relationship between potentiation and glucose levels. Relationship between the mean potentiation ratio and the mean glucose levels during the IIGI in NGT and T2D subjects (all doses pooled). In a bivariate linear regression model with the mean potentiation ratio as dependent variable, the slope of the relationship is significant in both groups (*P*<0.002) but flatter in T2D (*P*<0.0001 for the interaction term).

During IIGI in NGT subjects, the insulin secretion dose-response was shifted upwards at the two higher glucose doses, but with similar slopes (**Figure 1**). Thus, at a glucose level of 5.3 mmol/L, insulin secretion rate from the dose-response was 88 [33], 104 [70] and 126 [41] pmol^.^min^−1.^m^−2^ in the IIGI tests matching the 25, 75 and 125 g glucose dose, respectively (*P*<0.01). Rate sensitivity was low and dose-independent (*P* = 0.32) (**Table 1**). The mean potentiation ratio, on the other hand, increased with the glucose dose (*P*<0.0001 for the dose effect), and was positively related to insulin secretion at 5.3 mmol/L glucose (ρ = 0.63, *P* = 0.001, all doses pooled) and to mean glucose levels (ρ = 0.81, *P*<0.0001, all doses pooled; **Figure 2**). Thus, with i.v. glucose progressive stimulation by increasing glucose loads resulted in an insulin response characterized by stimulation of potentiation and an upward shift of the ß-cell dose-response, but comparable glucose sensitivity and early response.

During IIGI in patients with T2D, ß-cell function parameters showed a similar pattern as in NGT, *i.e.,* an upward shift of the dose-response (insulin secretion rate at 7.7 mmol/L glucose was 148 [77], 161 [105], and 184 [115] pmol^.^min^−1.^m^−2^ in the IIGI tests matching the 25, 75 and 125 g glucose dose, respectively, *P*<0.02) (Figure 1), dose-independent glucose sensitivity, virtually absent rate sensitivity, and only a trend for the mean potentiation ratio to increase with the dose (Table 1). As compared with subjects with NGT, ß-cell function was severely impaired, with null rate sensitivity and 60% reduced glucose sensitivity (*P*<0.0001 for the group effect). The mean potentiation ratio was directly related to mean glucose levels, but the relationship was flat compared to NGT (Figure 2).

### Incretin Effects (OGTT/IIGI Ratios)

**Figure 3:**
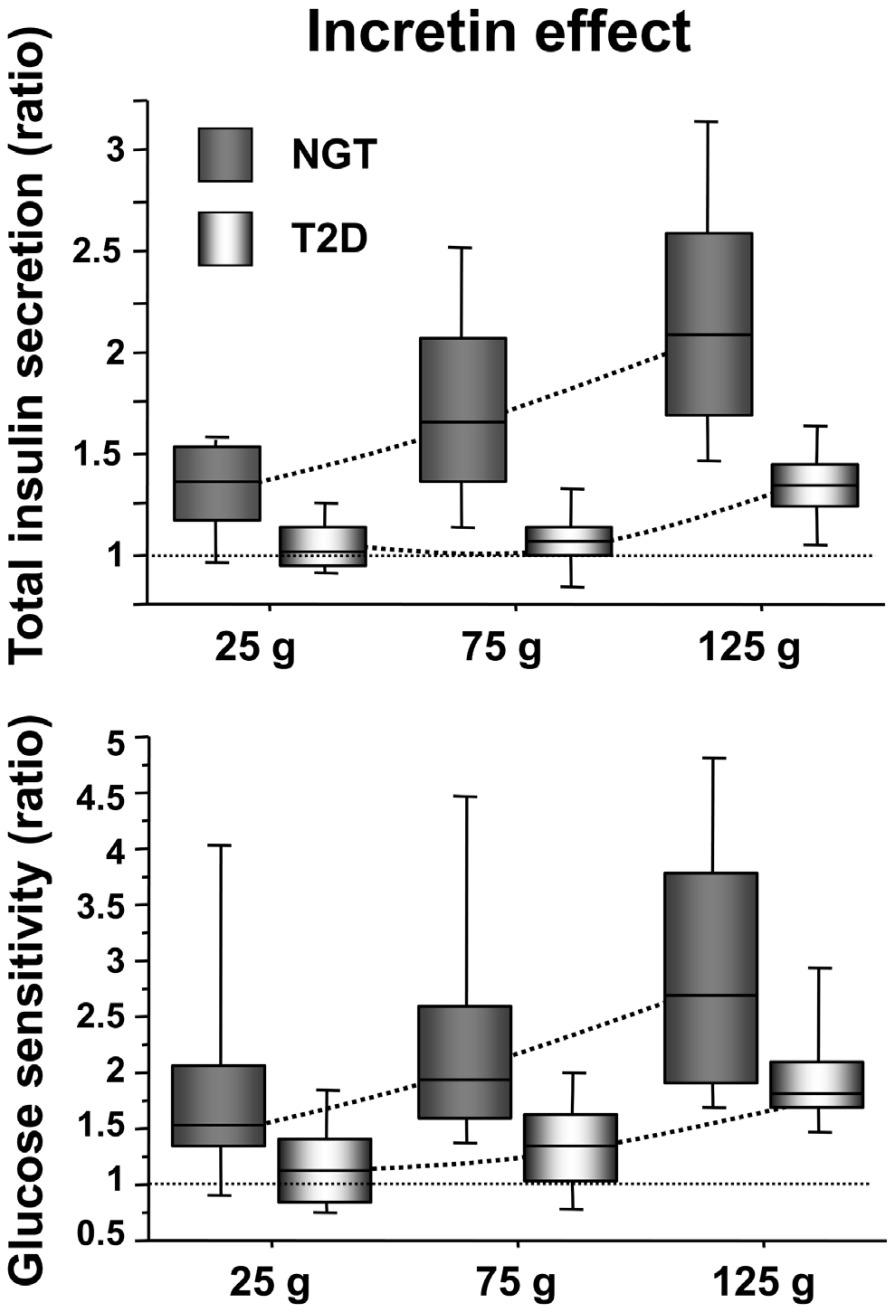
Incretin effect on total insulin secretion and glucose sensitivity. Incretin effect as the OGTT/IIGI ratio of total insulin secretion (*top panel*) and glucose sensitivity (*bottom panel*) in NGT and T2D subjects by glucose dose.

**Figure 4:**
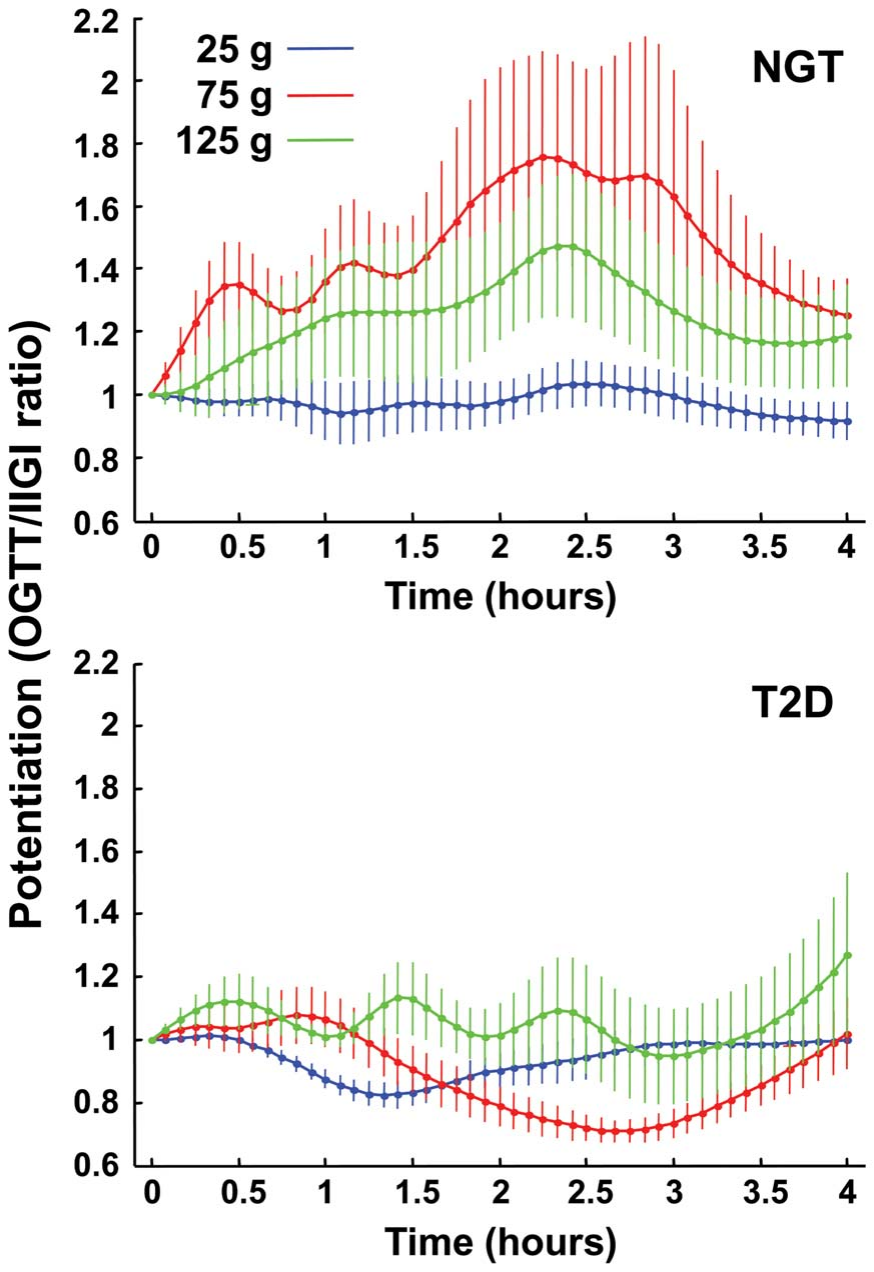
Incretin effect on potentiation. Time-course of the incretin effect on OGTT/IIGI ratio of the potentiation factor with increasing glucose loads in NGT and T2D participants. The potentiation factor is normalized to the baseline value before calculating the ratio. Plots are mean±SE.

In subjects with NGT, the incretin effect on total insulin secretion showed a remarkably wide range (0.87–3.3, all doses), increased progressively with the glucose load (Figure 3), and was significantly greater than 1 with all doses (*P*<0.0001 for the dose effect). Modulation of the incretin effect was mediated by a dose-dependent increase in glucose sensitivity (*P*<0.0001) (Figure 3), and a dose-independent enhancement of rate sensitivity *P*<0.0001, *P* = 0.07 for dose-dependence by Friedman test) (Table 1). The incretin effect on potentiation also increased dose-dependently (*P*<0.0001) (Figure 4).

In patients with T2D, the incretin effect on total insulin secretion demonstrated a narrower range as compared to NGT (0.78–1.7, all doses), which was significantly greater than 1 only at highest glucose load, and which was impaired as compared to NGT across doses (*P* = 0.004 for the group × dose effect) (Figure 3). The incretin effect on glucose sensitivity showed the same dose-dependence as in NGT, but was smaller than in the latter group across all 3 doses (*P* = 0.005) (Figure 3); rate sensitivity was stimulated (*P*<0.0001) in a dose-independent fashion (*P* = 0.42, Friedman test). In contrast to subjects with NGT, the incretin effect on potentiation was virtually absent (Figure 4), and significantly inferior to that of NGT (*P* = 0.015 for the group × dose interaction).

**Figure 5:**
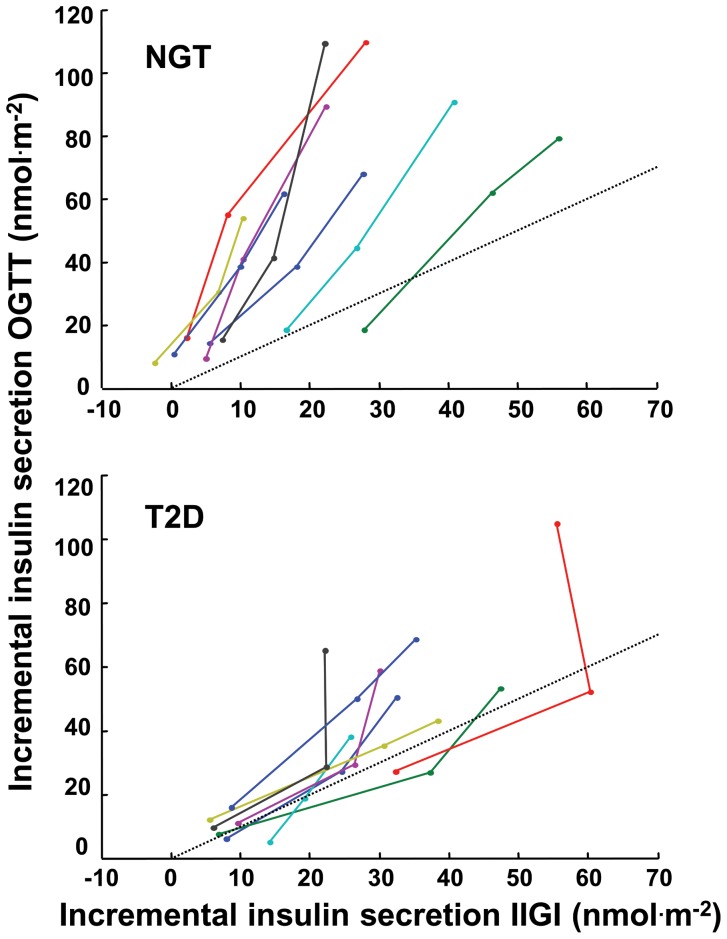
Incremental insulin secretion response to intravenous and oral glucose. Individual trajectories for the relationship between the dose-dependent increment above basal in total insulin secretion during the OGTT and the IIGI in NGT and T2D participants. The dotted lines are the identity line.

To further illustrate the mechanisms of the incretin effect, the increment above basal of insulin secretion during the OGTT was plotted against the corresponding increments during IIGI, at all doses (**Figure 5**). The individual relationships were remarkably linear in most cases, with some notable deviations in two T2D subjects. The slope of these lines was estimated by linear regression and denoted as incretin effect slope. In the two T2D subjects with a nonlinear pattern, this estimate does represent an average slope but is potentially less reliable. We have thus evaluated if the results were affect by the exclusion of these subjects. The incretin effect slope represents a dose-independent measure of the individual incretin effect: the steeper the line the greater the enhancement of oral vs IIGI glucose-induced increase in insulin secretion. Along the identity lines (dotted lines in Figure 5), the increments of insulin secretion during the i.v. and oral tests are the same, i.e., there is no incretin effect. The incretin effect slope, in contrast to the classical total incretin effect, is not intrinsically dependent on total insulin secretion and related parameters. The incretin effect slope was significantly impaired in patients with T2D (1.8 [0.8] vs 3.3 [1.2], *P* = 0.001; the significance was maintained after exclusion of the T2D subjects with potentially unreliable incretin effect slope).

### Incretin Hormone Responses

**Figure 6:**
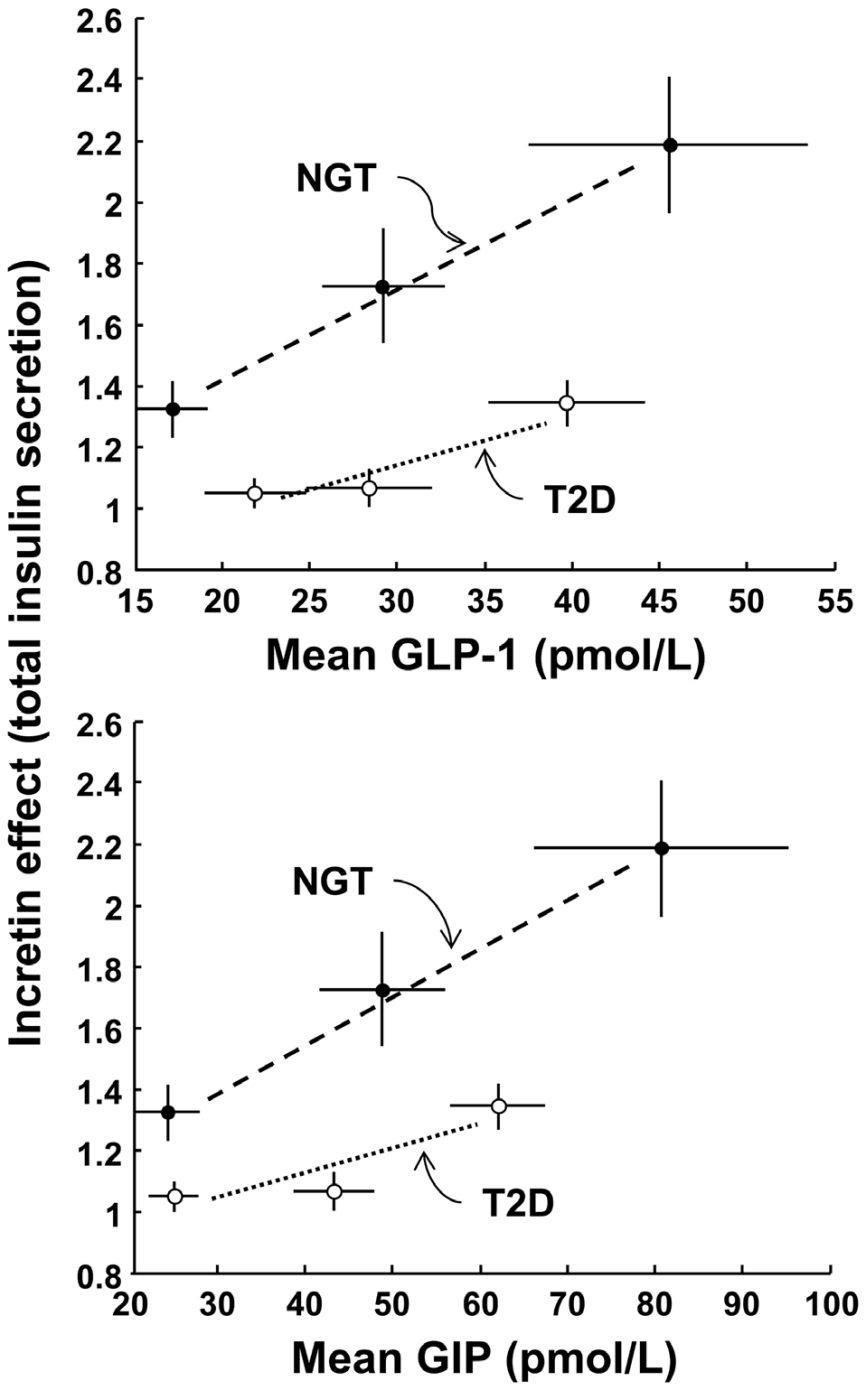
Average relationship between incretin effect and incretin hormones. Relationship between the incretin effect on total insulin secretion and the corresponding mean OGTT plasma GLP-1 and GIP in NGT and T2D participants. Plots are mean±SE.

**Figure 7:**
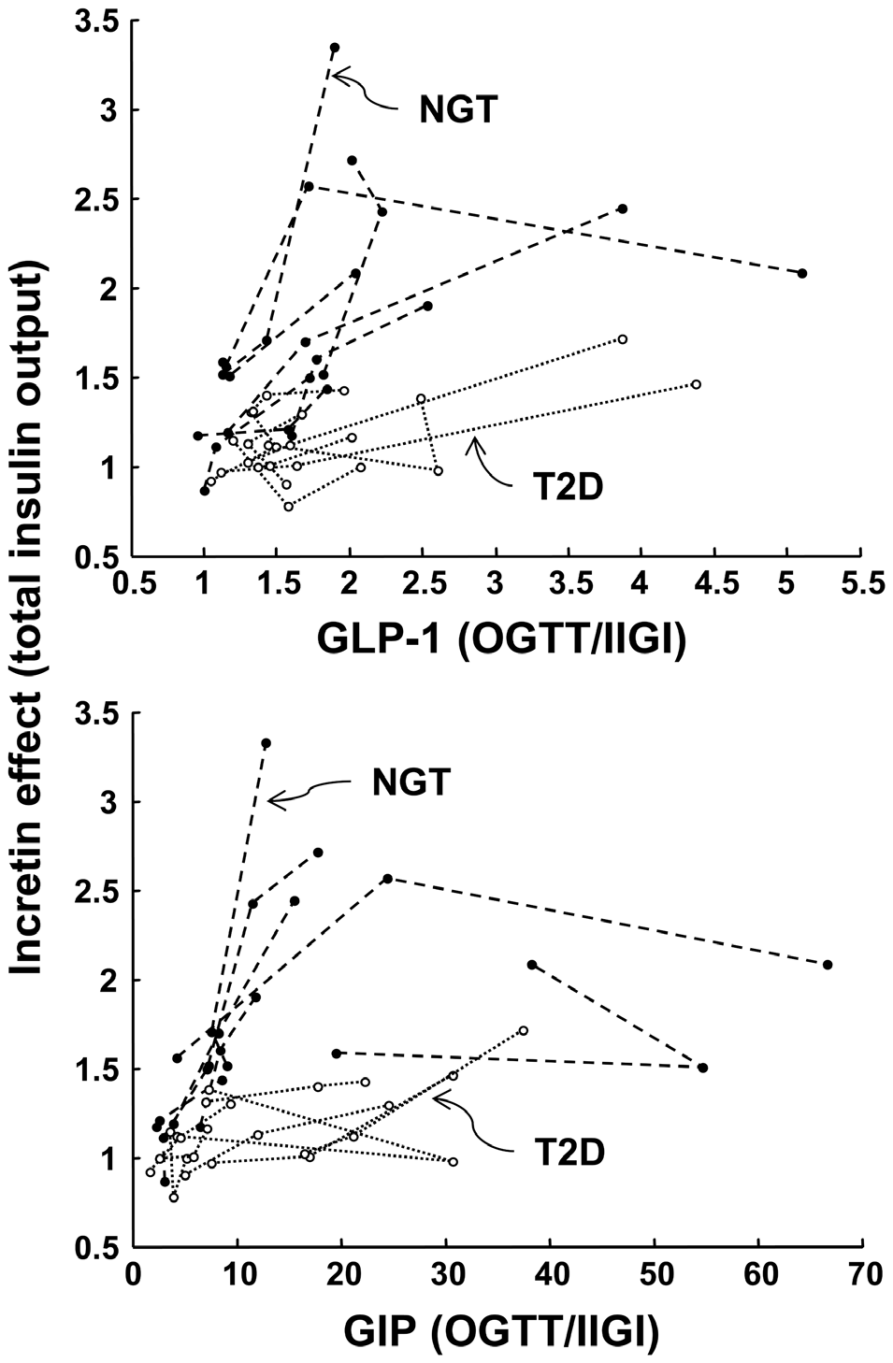
Individual relationships between incretin effect and incretin hormones. Individual trajectories for the relationship between the incretin effect on total insulin secretion and the mean OGTT/IIGI ratio of GLP-1 and GIP concentrations in NGT and T2D participants.

As previously reported [5], GLP-1 increased with the glucose dose during the OGTT but not during the IIGI (*P*<0.003 for the test × dose interaction) (Table 2). The responses were widely variable (especially for GIP), and differences in mean hormones between patients with NGT and T2D were not significant statistically. When the GLP-1 and GIP responses during the OGTT were regressed against the incretin effect on total insulin secretion (Figure 6), the relationship was significantly flatter in patients with T2D than subjects with NGT for both incretin hormones, as expected. Likewise, when the incretin effect on total insulin secretion was plotted against the OGTT/IIGI ratio of mean hormone concentrations, the individual dose trajectories for the patients with T2D ran consistently below those of the NGT individuals (Figure 7). Although the study didn’t allow rigorous assessment of the sensitivity to incretin hormones, an estimate could be obtained from the slope of the individual trajectories. The estimate of the sensitivity to GLP-1 in NGT was ∼3.5 fold that in patients with T2D; the estimate for GIP was ∼5 fold (NGT/T2D ratio of the median of the slopes; *P* = 0.02 for GLP-1 and *P*<0.05 for GIP).

Using the incretin effect slope (Figure 5), we assessed whether the magnitude of insulin secretion during the IIGI was related to the incretin effect. When all participants were pooled, the incretin effect slope was inversely related to the mean total insulin secretion at the three glucose doses during IIGI; the relationship was significantly shifted downwards in patients with T2D (*r* ^2^ = 0.66, *P* = 0.03 for total insulin secretion, *P*<0.02 for the group effect; the significance was maintained after exclusion of the T2D subjects with potentially unreliable incretin effect slope). A similar relationship was observed using fasting insulin secretion in place of the total insulin secretion.

### Insulin Sensitivity

The estimates of insulin sensitivity provided by the OGTT-based OGIS method were 380 [64], 383 [48], and 396 [58] ml^.^min^−1.^m^−2^ in NGT (*P* = ns for the dose effect), and 297 [39], 302 [37], and 316 [43] ml^.^min^−1.^m^−2^ in T2D (*P* = ns for the dose effect; *P*<0.0001 *vs* NGT).

### Determinants of Mean Glucose

In the whole group, mean glucose levels during the OGTT were very well predicted by the key variables of the study, i.e., the glucose dose, ß-cell glucose sensitivity from the IIGI, the total incretin effect, and insulin sensitivity. In a multiple linear regression model including these parameters (all log-transformed except for the glucose dose), the total explained variance was 93% (*P*≤0.01 for all parameters; standardized coefficients 0.63, −0.25, −0.42 and −0.43 for the glucose dose, insulin sensitivity, ß-cell glucose sensitivity and the incretin effect, respectively). In this model, the glucose tolerance status (NGT/T2D as categorical variable) was not a significant predictor (*P* = 0.27). A similar result was obtained when replacing ß-cell glucose sensitivity from the IIGI test and the incretin effect with glucose sensitivity from the OGTT (*r* ^2^ = 0.90, *P*<0.0005 for all predictors; standardized coefficients 0.62, −0.27 and −0.68 for the glucose dose, insulin sensitivity and ß-cell glucose sensitivity from OGTT, respectively).

## Discussion

Overall, the current data show that the incretin effect on all aspects of ß-cell function (total insulin secretion, ß-cell glucose sensitivity, rate sensitivity, and potentiation) is compromised in patient with T2D as compared with NGT subjects. This is consistent across a 5-fold increase in glucose load. The new modeling analysis of the original multiple dose study [5] has allowed us to extend the previous findings, which were mostly based on the traditional calculation of the incretin effect as the OGTT/IIGI ratio of total insulin secretion, and to address several specific questions of pathophysiological relevance.

The first question is whether the impaired incretin effect observed in hyperglycemic, insulin resistant conditions is due to saturation of ß-cell capacity, as suggested by Meier and Nauck [17]. Our results indicate that this is not the case. In fact, our multiple dose protocol shows that, not only in NGT but also in patients with T2D, during the IIGI the ß-cell responds to gradually increasing *infused* glucose loads with a progressive, commensurate increase in insulin secretion, such that the dose-response remains the same, or is even slightly shifted upward due to potentiation.

The second question is whether an impaired incretin effect reflects inherent ß-cell dysfunction. With the classical single-dose isoglycemic protocol, the incretin effect – as the OGTT/IIGI ratio of insulin secretion – is intrinsically correlated with insulin secretion itself, and therefore is a biased descriptor of the relationship between insulin secretion and ß-cell function. To address this problem, we have used the incretin effect *slope,* which is independent of glucose dose and insulin secretion. Calculation of this parameter was possible because the individual relationships between total incremental insulin secretion during the intravenous and the oral test were linear in most subjects (Figure 5). A significant deviation from linearity was observed in two T2D subjects, but whether this represents a feature of T2D or is only casual could not be decided from this small group. Nevertheless, we have found that the incretin effect slope is lower when fasting or IIGI-stimulated insulin secretion is higher regardless of diabetes status. Therefore, a lower incretin effect *per se* does not represent ß-cell dysfunction. In fact, in NGT subjects with a relatively lower incretin effect glucose sensitivity is preserved and the OGTT response is appropriate. The inverse relationship between incretin slope and IIGI-stimulated insulin secretion rather suggests that low secretion rates leave more “reserve” for the ß-cell to amplify insulin secretion when the incretin receptors are activated by oral glucose.

Thirdly, the multiple dose protocol has also allowed us to address the question of whether the incretin effect depends on the concentration of incretin hormones achieved during the OGTT (or their ratio to IIGI levels). As illustrated by our results in patients with T2D, the incretin effect on total insulin secretion was markedly depressed in T2D despite similar plasma levels of GLP-1 and GIP. Although our estimates of sensitivity to incretin hormones were obtained by simple regression analysis and with no possibility to distinguish between the role of GLP-1 and GIP, our results are compatible with the presence of resistance of ß-cells to GLP-1 and GIP in patients with T2D. This is in agreements with the findings of Kjems *et al.* [8], where increasing doses of exogenous GLP-1 were used in combination with a graded glucose infusion test to quantify GLP-1-induced increases in ß-cell glucose sensitivity.

With regard to the influence of the incretin mechanisms on different phases of insulin secretion, our current data show that the incretin effect on glucose sensitivity is dose-dependent, whereas the effect on early secretion, as assessed by the rate sensitivity parameter, is not. In patients with T2D, the incretin-mediated increase in rate sensitivity showed a similar dose pattern as in NGT, while glucose sensitivity was significantly enhanced only at the highest dose and the potentiation pattern was flat. This indicates that with the lowest glucose load (25 g) patients with T2D do respond to incretin stimulation, but only in the early phase of the insulin response. The degree of preservation of the incretin effect on the early insulin response in T2D cannot be quantified precisely, however. In subjects with NGT, the average OGTT/IIGI ratio for rate sensitivity was ∼2.5 fold, *i.e.*, of a similar magnitude as the incretin effect on total insulin secretion and glucose sensitivity, whereas in patients with T2D this ratio could not be calculated as rate sensitivity during the IIGI was virtually zero. It is interesting to speculate on the possible reasons of this finding. Activation of the GLP-1 and GIP receptors on the ß-cell produces a cascade of events leading to an increase of intracellular calcium levels, which directly stimulate exocytosis of insulin granules [18]. The calcium signal is essential for insulin secretion, but another crucial condition is the presence of insulin granules in close proximity to and ready to fuse with the cell membrane, with immediate release of insulin as a result. Thus, it is possible that in patients with T2D incretin hormones are able to produce the expected increase in calcium and to stimulate exocytosis, but the effects on secretion are transient because of lack of adequate granule supply (or *de novo* insulin biosynthesis).

In addition to the novel findings concerning the mechanisms of the incretin effects, this study clarifies some other relevant aspects of the ß-cell function. In subjects with NGT receiving i.v. glucose, an increase in mean glucose levels was accompanied by an enhancement of insulin secretion relative to glucose. This phenomenon was mediated by stimulation of potentiation which, from a flat pattern at the lowest dose (potentiation ratio equal to 1), shifts to the classical rising shape at the highest dose, as reported previously [15]. This dose-dependency of potentiation further supports the concept that exposure to higher glucose levels *per se* enhances ß-cell responsiveness [19] in a way that is time- and dose-dependent. In contrast to previous work, the unique feature of this study is that i.v. testing was performed at three mean glucose levels corresponding to those observed during the OGTTs. Forcing glucose to prescribed levels eliminates the known feedback by which stimulation of potentiation contributes to glucose lowering [20], and reveals for the first time a clear and strong relationship between glucose concentration and potentiation. Physiologically, this phenomenon does have quantitative relevance, as the increase in potentiation observed at the highest dose implies an increase in insulin secretion of ∼20% on average (and a considerably larger increase at the potentiation peak). The glucose- and time-dependent insulin secretion increase observed in this study with an OGTT-like glucose profile likely reflects the same phenomenon that generates the progressive insulin secretion rise seen during hyperglycemic clamps [21].

In patients with T2D the mechanisms underlying insulin secretion during i.v. testing were comparable to those observed in subjects with NGT, but ß-cell function was profoundly impaired in all respects. The defect in glucose sensitivity and rate sensitivity in T2D has been previously described [22], while the phenomenon of potentiation loss has not been described before. Notably, as hypothesized above for the incretin effect, a defective granule supply or recruitment process could be the common cause underlying these phenomena.

Using regression analysis on the 48 OGTTs, we have found that ß-cell glucose sensitivity from the intravenous test and the incretin effect were both important predictors of the mean glucose levels. Interestingly, these factors, in addition to insulin sensitivity and the glucose dose, explained a high proportion of the glucose variability, with no difference between NGT and T2D subjects. In this study we did not consider gastric emptying, as in the previous report [5], where it has been suggested that the progressive decrease of gastric emptying with the increasing glucose dose could have contributed to restrain glucose excursions. This hypothesis is not in contrast with the present regression model, in which the contribution of the glucose dose could be inclusive also of the effects on gastric emptying.

In conclusion, by modeling analysis of the relationship between glucose concentration and insulin secretion in a study employing ascending glucose doses, we have extended the investigation of the mechanisms of the incretin effect beyond the possibilities of the traditional method based on a single index. In particular, we have shown that in subjects with NGT the incretin effect on late-phase insulin secretion is progressive while that on early-phase secretion is not. In patients with T2D, the incretin effect on the late-phase insulin response is strongly impaired, while that on early-phase insulin response is partially preserved. The incretin effect is inversely related to insulin secretion levels at fasting and after i.v. glucose stimulation, and its relative impairment in subjects with NGT was not associated with a general ß-cell dysfunction.
